# Eighth Major Clade for Hepatitis Delta Virus

**DOI:** 10.3201/eid1209.060112

**Published:** 2006-09

**Authors:** Frédéric Le Gal, Elyanne Gault, Marie-Pierre Ripault, Jeanne Serpaggi, Jean-Claude Trinchet, Emmanuel Gordien, Paul Dény

**Affiliations:** * Hôpital Avicenne and EA3406, Université Paris 13, Bobigny, France;; † Hôpital Saint-Louis, Assistance Publique - Hôpitaux de Paris, Paris, France;; ‡ Hôpital Necker, Assistance Publique - Hôpitaux de Paris, Paris, France;; § Hôpital Jean Verdier, Assistance Publique - Hôpitaux de Paris, Bondy, France

**Keywords:** Hepatitis delta virus, Deltavirus genus, molecular epidemiology, phylogenetic analysis, emerging virus, chronic hepatitis, genotype, clade, dispatch

## Abstract

Hepatitis delta virus is the only representative of the *Deltavirus* genus, which consists of 7 differentiated major clades. In this study, an eighth clade was identified from 3 distinct strains. *Deltavirus* genetic variability should be considered for diagnostic purposes. Clinical consequences of the diversity have yet to be evaluated.

Hepatitis delta virus (HDV) is a subviral agent that can lead to severe acute and chronic forms of liver disease in association with hepatitis B virus. Delta hepatitis is highly endemic to several African countries, the Amazonian region, and the Middle East, while its prevalence is low in industrialized countries, except in the Mediterranean. The HDV genome is a circular, single-stranded RNA virus that ranges from 1,672 (strain dFr45, accession number *AX741144*) to 1,697 nucleotides (dFr47, *AX741149*) (*1*). A unique open reading frame encodes the small and large hepatitis delta (sHD and lHD, respectively) antigens by way of an editing step in the hepatocyte nucleus (*2*). Recent extensive analyses of HDV sequences from strains isolated from patients of African origin have shown a high genetic diversity of HDVs. To date, 7 major clades have been individualized with strong phylogenetic support; their proposed labels are HDV-1 to HDV-7 (*1*).

The genetic diversity of HDV is related to the geographic origin of the isolates. Apart from HDV-1, which is ubiquitous, each virus clade is geographically localized: HDV-2 (previously labeled HDV-IIa) is found in Japan (*3*), Taiwan (*4*), and Yakoutia, Russia (*5*); HDV-4 (previously labeled HDV-IIb) in Taiwan (*6*) and Japan (*7**,**8*); HDV-3 in the Amazonian region (*9*); and HDV-5, HDV-6, and HDV-7 in Africa. The eventuality of a genetic diversity extended to more than 7 clades has been mentioned by Radjef et al., who characterized a sequence (dFr644) that was not strongly affiliated to any of the 7 HDV clades (*1*). We describe 2 HDV isolates (dFr2072 and dFr2736) that have robust phylogenetic relation to dFr644 and, therefore, propose an extended classification of the *Deltavirus* genus to 8 clades.

## The Study

Strains dFr2072 and dFr2736 were isolated from 2 patients originating from Senegal and Côte d'Ivoire, respectively. The patients were living in France when chronic delta hepatitis was diagnosed; however, because no risk factors were identified, each patient was suspected to have been infected during childhood in Africa.

Full-length HDV genome sequences from isolates dFr2072 and dFr2736 were characterized to determine their genetic affiliation. HDV RNA extraction and cDNA synthesis were performed as previously described (*5*), and 4 overlapping regions of the genome were amplified (Table 1). Amplicons were sequenced bidirectionally with the BigDye Terminator v3.1 Sequencing Kit (Applied Biosystems, Courtabœuf, France).

**Table 1:** Four overlapping regions amplified by reverse transcription–PCR for full-length genome sequence determination

Region*	Primer name†	Primer position	Nucleotide sequence of the primers (5´–3´)
*R0* (889–1289)	889s	889–911	CATGCCGACCCGAAGAGGAAAG
	1289as	1289–1265	GAAGGAAAGGCCCTCGAGAACAAGA
*R´1* (305–1161)	305s	305–328	CTCCAGAGGACCCCTTCAGCGAAC
	1161as	1161–1138	CCCGCGGGTTGGGGATGTGAACCC
*R´2* (962–331)	962s	962–984	GTACACTCGAGGAGTGGAAGGCG
	331as	331–311	TCTGTTCGCTGAAGGGGTCCT
*R´3* (120–619)	120s	120–140	GTCCCAAGAGGGCGAGGGGAG
	620as	619–600	TCCTGGAGCCGGCAGTCCGG

*Name of the amplified region and position on the genome (according to Wang et al. [*10*]). †s, forward primer; as, reverse primer.

In a first approach, the complete dFr2072 and dFr2736 sequences were aligned with 41 complete genome sequences gathering all of the 7 HDV clades, plus sequence dFr644. The sequence alignment was generated in 2 ways: 1 with ClustalX using a gap-opening penalty (GOP) of 15 and a gap-extension penalty (GEP) of 6.66 and 1 with the SOAP program (http://evol-linux1.ulb.ac.be/ueg/SOAP/) (GOP from 12 to 17 in steps of 0.5; GEP from 6 to 8 in steps of 1). Phylogenetic analyses were performed with PAUP*4.0β10 (Sinauer Associates, Inc., Sunderland, MA, USA) from a SOAP sequence alignment that excluded 833 unstable characters. Neighbor-joining (NJ) distance and maximum parsimony (MP) analyses were performed. The robustness of the topologic features was determined by bootstrap methods (10^3^ replicates for NJ and MP). A Bayesian approach (*11*) was also used on the data matrix: 5,000 trees were initially built by using the MrBayes program, version 3.0 β4, from 2×10^6^ generations, and the first 250 trees were burned. A majority rule consensus tree was obtained by using PAUP*4.0 β10. Parameters specified during MrBayes analysis (Table 2) were also imported into PAUP*4.0 β10 for a maximum likelihood (ML) analysis using the general time reversible model with a gamma distribution.

**Table 2:** Parameters specified by MrBayes (version 3.0β4) application

Sequences	Substitution rate matrix*						Nucleotide frequencies				α†
	G/T	C/T	C/G	A/T	A/G	A/C	pi (A)	pi (C)	pi (G)	pi (T)	
Full length	1.000	2.733	0.681	1.344	3.011	0.847	0.200	0.304	0.288	0.208	0.526
sHD‡	1.000	6.687	0.926	2.221	3.550	1.519	0.322	0.216	0.361	0.101	0.434

*Each substitution rate is expressed as compared with the G/T substitution rate. †Parameter α is the shape parameter of the γ distribution. ‡Small hepatitis delta nucleotide sequence.

**Figure 1:**
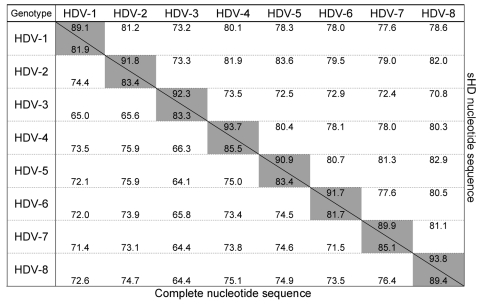
Percent similarity between hepatitis delta virus (HDV) genotypes calculated from complete and small hepatitis delta (sHD) nucleotide sequences. Above the oblique line are represented scores of similarity obtained from alignment and comparison of 49 sHD nucleotide sequences including 13 HDV-1 sequences, 7 HDV-2, 7 HDV-3, 6 HDV-4, 6 HDV-5, 4 HDV-6, 3 HDV-7, and 3 HDV-8. Below the oblique line are represented scores of similarity obtained from alignment and comparison of 44 complete nucleotide sequences including 13 HDV-1 sequences, 7 HDV-2, 4 HDV-3, 6 HDV-4, 6 HDV-5, 3 HDV-6, 2 HDV-7, and 3 HDV-8. Gray cells show the similarities within each genotype.

**Figure 2:**
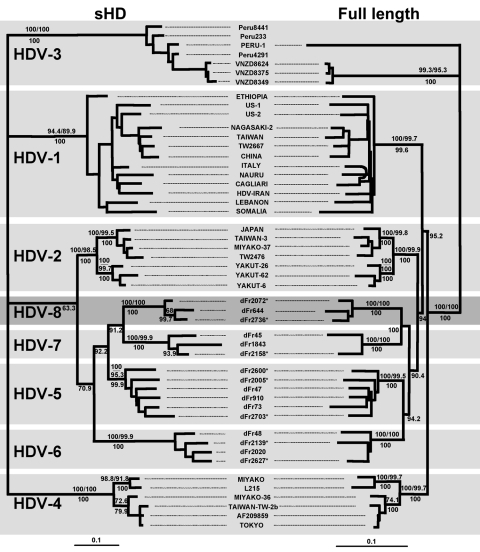
Maximum likelihood trees inferred from hepatitis delta virus (HDV) nucleotide sequences. Left panel: Maximum-likelihood phylogram obtained from the small hepatitis delta antigen dataset. Right panel: Maximum-likelihood phylogram obtained from the full-length HDV genome dataset. Bootstrap values (10^3^ replicates) obtained for neighbor-joining and maximum parsimony are indicated above the branches; posterior probabilities (inferred from 5×10^3^ trees generated from MrBayes application) are indicated below the branches. Asterisks indicate HDV sequences characterized in this study. Scale is in percent expected substitution per position. The accession numbers of the sequences used were AF209859, *AF209859*; Cagliari, *X85253*; China, *X77627*; dFr45, *AX741164*; dFr47, *AX741149*; dFr48, *AX741164*; dFr73, *AX741154*; dFr644, *AX741169*; dFr910, *AX741159*; dFr1843, *AJ583885*; dFr2005 (Guinea-Bissau), *AM183331*; dFr2020, *AJ583887*; dFr2072 (Senegal), *AM183330*; dFr2139 (Central African Republic), *AM183332*; dFr2158 (Cameroon), *AM183333*; dFr2600 (Togo), *AM183326*; dFr2627 (Nigeria), *AM183329*; dFr2703 (Senegal), *AM183328*; dFr2736 (Côte d'Ivoire), *AM183327*; Ethiopia, *U81989*; HDV-Iran, *AY633627*; Italy, *X04451*; Japan, *X60193*; L215, *AB088679*; Lebanon, *M84917*; Miyako, *AF309420*; Miyako-36, *AB118845*; Miyako-37, *AB118846*; Nagasaki-2, *AB118849*; Nauru, *M58629*; Peru-1, *L22063*; Somalia, *U81988*; Taiwan, *M92448*; Taiwan-3, *U19598*; Taiwan-Tw-2b, *AF018077*; Tokyo, *AB118847*; TW2476, *AF104264*; TW2667, *AF104263*; US-1, *D01075*; US-2, *L22066*; Vnzd8349, *AB037948*; Vnzd8375, *AB037947*; Vnzd8624, *AB037949*; Yakut-26, *AJ309879*; and Yakut-62, *AJ309880*.

An 89.4% similarity score was observed between dFr644, dFr2072, and dFr2736, which allowed these 3 sequences to be grouped inside the same genotype. By contrast, the similarity obtained among dFr644, dFr2072, and dFr2736 and the sequences representative of HDV-1 to HDV-7 was <76.4% (Figure 1). On the phylogenetic tree built from the ML data (Figure 2), isolates dFr644, dFr2072, and dFr2736 appeared as a monophyletic group, with bootstrap values of 100 (NJ and MP) and a posterior probability value of 100 (MrBayes).

Because of claims that the sHD protein trans-complements the corresponding HDV type more efficiently (*12*), we compared the sHD coding nucleotide sequences of dFr644, dFr2072, and dFr2736 with 46 sequences, by using the same phylogenetic approaches (Table 2). Analysis of the sHD genes confirmed the results obtained with the full-length sequences, showing 93.8% similarity between dFr644, dFr2072, and dFr2736 versus only 70.8%–82.9% when compared with sequences of the other genotypes (Figure 1). Bootstrap values of 100 (NJ and MP) and posterior probability values of 100 (MrBayes) were obtained and are represented on the phylogenetic tree built from the ML parameters (Figure 2). Taken together, these results fulfill the recommendations for the designation of a major clade (i.e., >3 distinct isolates repeatedly showing high scores of similarity and high bootstrap values [13]). Thus, we define an eighth major clade among the *Deltavirus* genus.

## Conclusions

In this study, an eighth HDV clade (HDV-8) was identified from 3 complete sequences obtained from strains isolated from patients of African origin. Isolate dFr644, originating from Congo-Brazzaville, was initially described by Radjef et al. and tentatively affiliated with HDV-7 (bootstrap value 84, posterior probability value 97), despite a similarity of only 77.8% with the other HDV-7 sequences (*1*). Isolates dFr2072 and dFr2736 presented similarity of 89.4% with dFr644 and only 76.4% with HDV-7 sequences. Thus, an additional lineage was individualized, bringing the number of HDV clades with a probable African origin to 4.

Since 1999, a total of 468 HDV isolates collected in France were analyzed in our laboratory for phylogenetic characterization of the *R0* region (defined in Table 1). Of these, 98 isolates (21%) were affiliated with HDV-5 (15.2%), HDV-6 (1.7%), HDV-7 (3.0%), or HDV-8 (1.1%) (Paul Dény, unpub data). The 98 corresponding patients were all of African origin. By contrast, all patients of European origin were specifically infected by HDV-1 isolates. To date, no evidence exists that HDV-5, -6, -7 or -8 circulates among native populations in France. These results strongly suggest the African origin of these viruses. Nevertheless, epidemiologic studies in Africa should be carried out to specify the prevalence and geographic distribution of all HDV clades. If the African origin of HDV-5, -6, -7 and -8 viruses is confirmed, detection of these clades in France among local populations would reveal an emerging process that should be anticipated in epidemiologic surveys. Thus, the molecular assays used for diagnostic purposes should rely on primers and probes defined in the most conserved regions of the HDV genome to avoid false-negative results (*5**,**14**,**15*).

In conclusion, the *Deltavirus* genus includes at least 8 major clades, with specific geographic distribution. Future development of molecular assays for diagnosis of delta hepatitis should take into account this high genetic variability. The relationship between HDV diversity and pathogenesis has previously been suggested (*7**,**9*) but remains to be clarified by taking into account the extension of the diversity. Treatment of chronic delta hepatitis, which relies on long-term administration of high doses of interferon-alpha, is not very effective (*16*). It is not known whether some HDV genotypes might be more susceptible to therapy than others, as has been described for chronic hepatitis C (*17*). Thus, the clinical effect of HDV diversity, in terms of severity of disease and response to therapy, remains to be determined.
